# Antibiotic susceptibility profiles among *Campylobacter* isolates obtained from international travelers between 2007 and 2014

**DOI:** 10.1007/s10096-017-3032-6

**Published:** 2017-06-17

**Authors:** A. Post, D. Martiny, N. van Waterschoot, M. Hallin, U. Maniewski, E. Bottieau, M. Van Esbroeck, E. Vlieghe, S. Ombelet, O. Vandenberg, J. Jacobs

**Affiliations:** 1 Department of Clinical Sciences, Institute of Tropical Medicine, Nationalestraat 155, 2000 Antwerp, Belgium; 2 National Reference Centre for Campylobacter, CHU Saint-Pierre, Brussels, Belgium; 3 Department of Microbiology, LHUB-ULB, Pôle Hospitalier Universitaire de Bruxelles, Brussels, Belgium; 4 Faculté de Médecine et Pharmacie, Université de Mons, Mons, Belgium; 5 Faculty of Health, Medicine and Life Sciences, Maastricht University, Maastricht, the Netherlands; 6 Department of Molecular Biology, LHUB-ULB, Pôle Hospitalier Universitaire de Bruxelles, Brussels, Belgium; 7 Department of General Internal Medicine, Infectious Diseases and Tropical Medicine, University Hospital Antwerp, Antwerp, Belgium; 8 University of Antwerp, Antwerp, Belgium; 9 Department of Microbiology and Immunology, KU Leuven, Leuven, Belgium; 10 Center for Environmental Health and Occupational Health, School of Public Health, Université Libre de Bruxelles, Brussels, Belgium

## Abstract

*Campylobacter* infection is a common cause of diarrhea among international travelers. We studied antibiotic resistance patterns among *Campylobacter* isolates obtained from international travelers according to travel destination. Three collections of isolates obtained from international travelers between 2007 and 2014 (Institute of Tropical Medicine, the “Laboratoire Hospitalier Universitaire de Bruxelles “and the Belgian National Reference Centre for *Campylobacter*) were used. Isolates were tested for minimal inhibitory concentration (MIC) values (E-test macromethod) for fluoroquinolones, macrolides, tetracyclines, amoxicillin–clavulanic acid, and meropenem. Single isolates from 261 travelers were available; median (IQR) age was 25.4 (4–42) years, 85.8% were symptomatic (information for 224 patients available). Overall resistance to ciprofloxacin was 60.9%, ranging from 50.8% in Africa to 75.0% in Asia. Resistance to erythromycin was 4.6%, with the highest rate observed for Southern Asia (15.2%, seven isolates, six of them recovered from patients returning from India). A total of 126 isolates (48.3%) were resistant to tetracycline. No resistance to amoxicillin–clavulanic acid or meropenem was detected. Ciprofloxacin resistance tended to increase over time (53.9% in 2007 versus 72.2% in 2014), erythromycin resistance remained stable (median annual resistance 4.2%). Most (86.2%) ciprofloxacin-resistant isolates had MIC values ≥32 mg/l, and all erythromycin-resistant isolates had MIC values ≥256 mg/l. Co-resistance to ciprofloxacin and erythromycin was observed in 11 (4.2%) isolates, seven of which came from Southern Asia. Among all regions of travel, more than half of *Campylobacter* isolates were resistant to ciprofloxacin. Overall resistance to erythromycin was below 5% but reached 15.2% in Southern Asia.

**Electronic supplementary material:**

The online version of this article (doi:10.1007/s10096-017-3032-6) contains supplementary material, which is available to authorized users.

## Introduction

*Campylobacter* spp., in particular *Campylobacter jejuni* and *Campylobacter coli*, are the most common cause of bacterial enteritis in humans worldwide. Typically *Campylobacter* infection causes self-limiting diarrhea with or without fever [1]. The severity of symptoms may require antibiotic treatment; most commonly erythromycin or fluoroquinolones [1–3]. In industrialized countries, travel-related campylobacteriosis represents an important subset of all reported cases, ranging from 13% in the USA [4] to 20% in Scandinavia [5, 6].

The objective of the present study was to update antibiotic resistance rates among *Campylobacter* isolates obtained from international travelers between 2007 and 2014, compared to a previous study over the time period 1994–2006 [7].

## Methods

### Design

Three collections of human intestinal *Campylobacter* isolates consecutively obtained from international travelers were tested. A first collection one (*n* = 194) was obtained from the post-travel policlinic at the Institute of Tropical Medicine (ITM) Antwerp. The second collection (*n* = 84) consisted of clinical isolates obtained from the Laboratoire de la Porte de Hal (LHUB-ULB), Brussels. The third collection (*n* = 37) formed part of a National Survey in Belgium, performed by the National Reference Centre for Campylobacter in Brussels (NRC). Methods of isolation and identification of isolates differed between collections. A detailed description of patient population, study period, and methods used for each collection can be found in Supplement 1. Only patients with a history of travel (≤2 weeks if symptomatic and ≤8 weeks if asymptomatic) prior to consultation were included in this study, and only the first isolate per patient was considered. For the purpose of this study, subcultures of all isolates obtained at LHUB-ULB or NRC from patients with a reported travel history were shipped to ITM and stored on Microbank at −80 °C pending testing.

### Antibiotic susceptibility testing

Upon retrieval from storage isolates were subcultured onto Columbia agar with 5% sheep blood and incubated at 42 °C for 24 h. Antibiotic susceptibility testing was performed in batch using Mueller Hinton agar with 5% sheep blood and incubated in a microaerophilic atmosphere at 42 °C for 24 to 48 h. Minimal inhibitory concentration (MIC) values for ciprofloxacin, levofloxacin, azithromycin, erythromycin, tetracycline, doxycycline, amoxicillin–clavulanic acid and meropenem were determined by E-test macromethod (bioMérieux, Marcy-l’Etoile, France). This panel was chosen in accordance with EUCAST guidelines and recommendations of the European Centre for Disease Prevention and Control (ECDC) [8]. Breakpoints are summarized in Table 1. EUCAST does not provide breakpoints for amoxicillin-clavulanic acid and levofloxacin. For amoxicillin–clavulanic acid, the EUCAST breakpoints for *Enterobacteriaceae* were used. For levofloxacin EUCAST breakpoints for *Enterobacteriaceae* are the same as those proposed for *Helicobacter pylori*, which is often considered as a *Campylobacter*-related organism [9]. For meropenem, the interpretation criteria as recommended by the Clinical and Laboratory Standards Institute (CLSI) were used [8, 10]. Quality control for antibiotic susceptibility was done according to manufacturer’s guidelines.

**Table 1:** Panel of tested antibiotics and corresponding breakpoints

Antibiotic	Breakpoint* (mg/l)	Reference guideline
Ciprofloxacin	≤0.5	EUCAST guideline for *Campylobacter*
Levofloxacin	≤1	EUCAST guideline for *Helicobacter pylori*
Erythromycin		EUCAST guideline for *Campylobacter*
*C. jejuni*	≤4	
*C. coli*	≤8	
Azithromycin	None	EUCAST and CLSI mention to use results for erythromycin to determine susceptibility to azithromycin
Tetracycline	≤2	EUCAST guideline for *Campylobacter*
Doxycycline	None	No breakpoints available, EUCAST and CLSI mention to use results for tetracycline to determine susceptibility to doxycycline
Amoxicillin/clavulanic acid	≤8	EUCAST guideline for *Enterobacteriaceae*
Meropenem	<16	No breakpoints available, EUCAST mention to use CLSI guideline for *Campylobacter*

*S and R breakpoints are identical

Guidelines: EUCAST guideline version 7.0, 2017 / CLSI guideline M100-S26, 2016

### Demographic and clinical data

Travel destinations were grouped in regions and continents according to the United Nations geoscheme [11]. Demographic and clinical data including age, gender, fever, diarrhea, and duration of symptoms were retrieved from patient files or from forms filled by participating labs and recorded in a protected database. Data were entered into an encrypted database for analysis.

### Statistical analysis

Proportions of resistance rates over time and for different geographical regions were assessed for significance using the Pearson’s chi-square test (X^2^-test) or Fisher’s exact test. Trends over time were assessed using the Mantel–Haenszel extension of the chi-square test for trends. A *p*-value <0.05 was considered significant. For convenience, differences between the three collections are only discussed separately in the text if they are significant.

### Ethical approval

Ethical approval was obtained at the respective ethics committees for both study sites; the Internal Review Board and the University Hospital of Antwerp for ITM and Ethical Committee of CHU Saint Pierre.

## Results

Of the 267 available isolates, six were excluded because information about travel destination was not available, resulting in a total of 261 isolates recovered from 261 patients. At ITM, 142 isolates were available for analysis, representing 73.2% of all 194 non-duplicate isolates obtained at this site during the study period: non-available isolates included those that had not been stored (*n* = 41) and those that did not grow upon retrieval (*n* = 11). The LHUB-ULB collection consisted of 83 available isolates, representing 98.8% of 84 isolates from patients with a recent travel history. In addition, 36 (97.3% of 37) isolates from participants with a recent travel history were available through the National Survey. *C. jejuni* was most prevalent (*n* = 230, 88.1%), followed by *C. coli* (*n* = 22, 8.4%); nine isolates (3.4%) were not identified beyond the genus level. There was no significant difference in geographical distribution between the different *Campylobacter* species.

### Patient characteristics and geographical origin of isolates

The median (IQR) age of the entire cohort was 25.4 (4–42) years; 50.4% of patients were male. Median ages differed between the three collections: 28.5, 2.5, and 49.0 years for the ITM, LHUB-ULB and National Survey collections respectively, with 8.4%, 58.3%, and 16.7% of patients younger than 5 years respectively. Complete data about a history of diarrhea and fever were available for 243 (93.1%) and 249 (95.4%) patients respectively: most (224, 85.8%) suffered from diarrhea, whereas 88 (33.7%) reported fever and 84 (31.8%) reported both. Seventeen patients were asymptomatic; in these participants, the isolate was obtained during routine post-travel consultation. Table 2 presents the distribution of travel destinations of the patients with *Campylobacter* infection, grouped according to the United Nations geoscheme [11]. More than three-quarters of travelers had returned from Africa (46.0%) or Asia (30.7%).

**Table 2:** Travel destinations of patients with a *Campylobacter* infection, represented per patient group. In brackets, countries with high numbers of isolates). Total number of isolates = 261

Travel destination according to UN geoscheme	Most frequently visited countries in the region (number of isolates)	ITM, *n* = 142	LHUB-ULB, *n* = 83	National survey, *n* = 36	Total, *n* = 261
		*N* (%)	*N* (%)	*N* (%)	*N* (%)
Eastern Africa	Ethiopia (12), Tanzania (5)	26 (18.3%)	3 (3.6%)	1 (2.8%)	30 (11.5%)
Middle Africa	DRC^1^ (16)	18 (12.7%)	7 (8.4%)	0	25 (9.6%)
Northern Africa	Morocco (22), Tunis (5)	5 (3.52%)	24 (28.9%)	7 (19.4%)	36 (13.8%)
Southern Africa	South Africa (1)	1 (0.7%)	0	0	1 (0.4%)
Western Africa	Burkina Faso (6), Cameroon (5)	20 (14.1%)	8 (9.6%)	0	28 (10.7%)
Africa total		70 (49.3%)	42 (50.6%)	8 (22.2%)	120 (46.0%)
Caribbean and Latin America	Cuba (1)	2 (1.4%)	1 (1.2%)	0	3 (1.1%)
Central America	Nicaragua (1)	1 (0.7%)	0	0	1 (0.4%)
South America	Peru (9)	13 (9.2%)	2 (2.4%)	0	15 (5.7%)
Northern America	USA^2^ (1)	0	0	1 (2.8%)	1 (0.4%)
America total		16 (11.3%)	3 (3.6%)	1 (2.8%)	20 (7.7%)
Eastern Asia	China (1)	1 (0.7%)	0	0	1 (0.4%)
Southern Asia	India (31), Nepal (5), Pakistan (5)	31 (21.8%)	13 (15.7%)	2 (5.6%)	46 (17.6%)
Southeastern Asia	Indonesia (10)	21 (14.8%)	1 (1.2%)	3 (8.3%)	25 (9.6%)
Western Asia	Turkey (7)	0	2 (2.4%)	6 (16.7%)	8 (3.1%)
Asia total		53 (37.3%)	16 (19.3%)	11 (30.6%)	80 (30.7%)
Eastern Europe	Poland (3), Romania (2)	0	6 (7.2%)	2 (5.6%)	8 (3.1%)
Southern Europe	Spain (8)	2 (1.4%)	9 (10.8%)	6 (16.7%)	17 (6.5%)
Western Europe	France (10)	1 (0.7%)	7 (8.4%)	8 (22.2%)	16 (6.1%)
Europe total		3 (2.8%)	22 (26.5%)	16 (44.4%)	41 (15.7%)

1. DRC: Democratic Republic of the Congo

2. USA: United States of America

### Antibiotic resistance: Geographic distribution and evolution over time (Table 3)

A total of 77 isolates (29.5%) were susceptible to all antibiotics tested, of which 43 isolates (55.8%) were obtained from Africa. Overall resistance to ciprofloxacin was 60.9%, ranging from 50.8% in Africa to 75.0% in Asia. Lowest resistance rates — although consistently above 33% of isolates — were observed for Eastern, Western, and Middle Africa. Overall resistance to erythromycin was 4.6%, and ranged from 2.4% in Europe to 10.0% in Asia. The highest rate was observed for Southern Asia (15.2%), with 6/7 resistant isolates recovered from India. A total of 126 isolates (48.3%) were resistant to tetracycline, with small variations between regions. There was no resistance to amoxicillin—clavulanic acid or meropenem. During the study period, ciprofloxacin resistance rate was higher in 2014 (72.2%) than in 2007 (53.9%). When comparing the present ciprofloxacin resistance rate to that of 2001–2006 as tested in our previous study [7],we find a significant increase over time (*p* = 0.03).

**Table 3:** Resistance rates of *Campylobacter* isolates represented by travel region defined according to United Nations geoscheme. Intermediate susceptible results are grouped together with resistant results

Region	*N*	Ciprofloxacin	Erythromycin	Tetracycline
Eastern Africa	30	13 (43.3%)	0	17 (56.7%)
Middle Africa	25	13 (52.0%)	1 (4.0%)	12 (48.0%)
Northern Africa	36	24 (66.7%)	1 (2.8%)	18 (50.0%)
Southern Africa	1	0	0	0
Western Africa	28	11 (39.3%)	1 (3.6%)	9 (32.1%)
Africa total	120	61 (50.8%)	3 (2.5%)	56 (46.7%)
Caribbean	3	1	1	0
Central America	1	1	0	1
South America	15	12 (80%)	0	9 (60.0%)
Northern America	1	1	0	1
America total	20	15 (75.0%)	1 (5.0%)	11 (55.0%)
Eastern Asia	1	0	0	0
Southern Asia	46	35 (76.1%)	7 (15.2%)	19 (41.3%)
Southeastern Asia	25	17 (68.0%)	1 (4.0%)	14 (56.0%)
Western Asia	8	8	0	4
Asia total	80	60 (75.0%)	8 (10.0%)	37 (46.3%)
Eastern Europe	8	4	0	6
Southern Europe	17	8 (47.1%)	0	6 (35.3%)
Western Europe	16	11 (68.8%)	1 (6.3%)	10 (62.5%)
Europe total	41	23 (56.1%)	1 (2.4%)	22 (53.7%)
Total	261	159 (60.9%)	12 (4.6%)	126 (48.3%)

If the total number of isolates for a particular region is <10, the percentage of resistance is not mentioned.

**Fig. 1:**
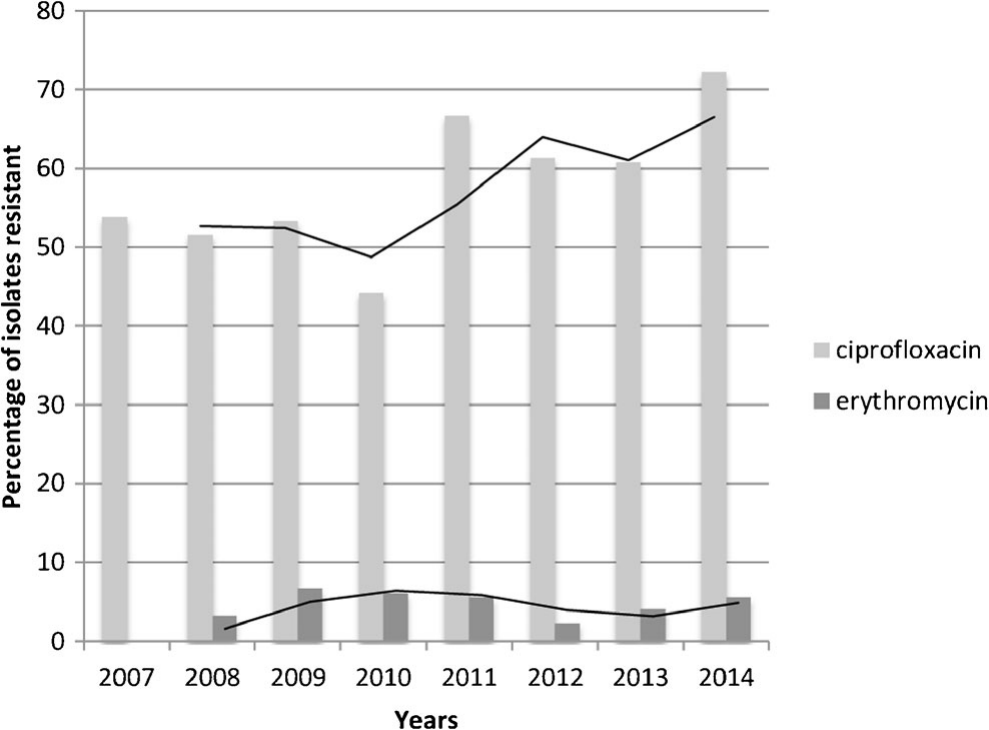
Evolution of ciprofloxacin and erythromycin resistance (expressed as % resistance) over the time period 2007–2014

For erythromycin, no increase over time was noted (median annual resistance 4.2%, Fig. 1). Compared to the aforementioned study [7], overall resistance against erythromycin was slightly, but not statistically higher in the present study compared to the 2001–2006 period (4.6% versus 2.7%, *p* = 0.2).

### Co-resistance and MIC values

**Fig. 2:**
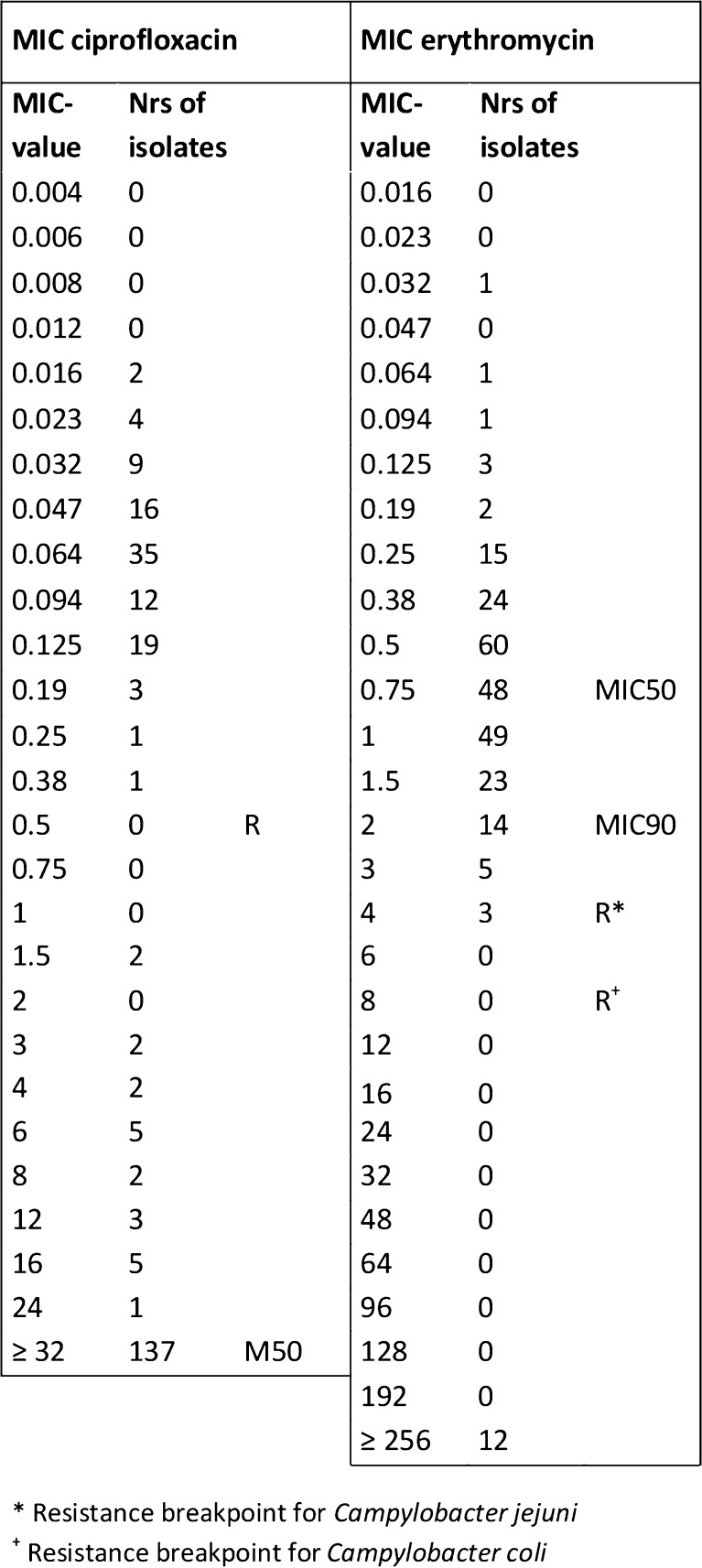
MIC-value distribution including MIC50 and MIC90 for ciprofloxacin and erythromycin among *Campylobacter* isolates, recovered from international travelers. *R* displays resistance breakpoint (see Table 1)

Resistance rates for levofloxacin and ciprofloxacin were identical (60.9%, 159 isolates) with complete overlap. Most (86.2%) ciprofloxacin-resistant isolates (86.2%) had MIC values ≥32 mg/l (Fig. 2). Levofloxacin-resistant strains displayed two subgroups; isolates with lower MIC values (around 6 mg/l, 60.0% of resistant isolates) and isolates with high MIC values (≥32 mg/l, 34.4% of resistant isolates). Resistance to erythromycin and azithromycin was seen among 12 isolates (4.6%); MIC values were in complete overlap, and all isolates displayed high-level resistance (≥256 mg/l) for both antibiotics. Resistance rates to erythromycin among *Campylobacter coli* tended to be lower than to those for *Campylobacter jejuni;* 9.1% and 4.3% respectively (*p* = 0.3). Resistance rates to ciprofloxacin were similar for both species. Co-resistance to ciprofloxacin and erythromycin was observed in 11 (4.2%) isolates, seven of which came from Southern Asia (six from India, one from Nepal).

## Discussion

In this study, we assessed antimicrobial resistance patterns among *Campylobacter* isolates obtained from stool samples from international travelers between 2007 and 2014.

The isolates used in this study came from collections with different travelers’ profiles and travel destinations: ITM serves a large patient population returning or originating from sub-Saharan Africa, while the LHUB-ULB serves hospitals that provide healthcare to a large population of Northern African origin. The National Survey was conducted during the holiday season and is likely to be the best representation of the Belgian tourists’ destinations (Europe and Asia). Most travelers were symptomatic.

### Comparison to literature

Overall, more than half of *Campylobacter* isolates were resistant to ciprofloxacin, with highest rates observed for Asia and the Americas. Data about resistance to fluoroquinolones among *Campylobacter* spp. in humans varies, with levels as high as 93.1% in China [12] to 6.6% in Finland [6]. The increase in ciprofloxacin resistance over the present study period is in line with what we observed previously [7], and was mainly accounted for by a steady increase in resistance among the African regions.

More than 80% of ciprofloxacin-resistant isolates had MIC values ≥32 mg/l. As 32 mg/l is the upper value at which the E-test is truncated, the actual MIC value of these isolates was not assessed. It should be remembered that peak concentration of fluoroquinolones in feces largely exceeds those reached in blood after standard-dose therapy. This can explain why fluoroquinolone treatment of campylobacteriosis may result in clinical cure despite apparent resistance of the isolate (23). Molecular mechanisms for fluoroquinolone resistance (not assessed in the current collection) include point mutations in the genes encoding DNA gyrase (*gyr*A) and an efflux system mechanism [13–15].

Resistance to macrolides as assessed in the present study was particularly high in Asia, with a resistance rate of 15.2% in Southern Asia. These results confirm the high level of resistance previously reported in a Pakistani urban environment in 2012, where 27.0% of *C. jejuni* human isolates were resistant to erythromycin [16]. Resistance to macrolides has been reported to be higher among *C. coli* (up to 66% [17]) compared to *C. jejuni* (less than 10%), but this was not confirmed in the present study, possibly because of low numbers of *C. coli* isolates.

The 15.2% resistance rate among isolates from Southern Asia, as well as the occurrence of combined ciprofloxacin–erythromycin resistance in India and Nepal (representing 7/11 co-resistant isolates), argue for caution and continued surveillance. Resistant isolates displayed high-level resistance for both antibiotics (MIC value ≥256 mg/l). This is indicative of resistance conferred by point mutations in the domain V in the 23S ribosomal RNA gene [17]. Our results confirm that azithromycin susceptibility can indeed be derived from erythromycin results.

In the present collection, there was no resistance against amoxicillin–clavulanic acid. Data about resistance levels to amoxicillin–clavulanic acid are scarce, as it is currently not a treatment of choice [18]. Resistance to carbapenems was not observed in the present study, but has been reported anecdotally among *Campylobacter fetus* [19].

### Limitations

The study was conducted with different isolate collections, originating from different time periods and various methods of isolation and identification. Additionally, the numbers of isolates varied between years, and 32 isolates obtained at ITM between October 2010 and August 2011 had not been stored. Detailed and complete clinical data (symptoms, duration of illness) were only available for a subset of patients. Finally, the number of isolates obtained from some regions were too small to draw reliable conclusions.

Conversely, combining different isolate collections made it possible to cover different profiles and destinations of travelers; the resulting numbers of isolates were sufficient to generate a geographic picture as well as an over-time evolution. In that regard, the study can be considered as representative for travel medicine, and in addition provides proxy antibiotic surveillance data from regions in the world where microbiological surveillance is scarce.

### Clinical implications

Antibiotic treatment for *Campylobacter* diarrhea is rarely indicated, as it is self-limiting and will usually cure within 5–7 days of onset of symptoms. In the case of patients whose symptoms are severe or persistent, immunocompromised patients, and in those with extra-intestinal infections, antibiotic treatment is needed. The present resistance rates confirm that, if indicated, azithromycin is the antibiotic of choice in travel-associated diarrhea contracted worldwide [7].

In conclusion, more than half of *Campylobacter* isolates recovered from symptomatic travelers returning from all regions were resistant to ciprofloxacin, precluding its use in the treatment of *Campylobacter* diarrhea. Overall resistance to erythromycin was below 5%, but as high as 15.2% in Southern Asia. The World Health Organization recently ranked fluoroquinolone-resistant *Campylobacter* spp. as a high-priority pathogen for the development of new antibiotics [20]. This necessity is highlighted by the increase in combined fluoroquinolone and macrolide resistance in Southern Asia, as demonstrated in this study. This study furthermore underlines the importance of routine surveillance, in particular in low-resource settings, and the reinforcement of restrictions on the use of antibiotics in humans and the animal industry.

## Electronic supplementary material

ESM 1 (DOCX 14 kb)

ESM 2 (XLSX 60 kb)
