# Supplementary material for: Antibiotic susceptibility profiles among Campylobacter isolates obtained from international travelers between 2007 and 2014
Source: Eur J Clin Microbiol Infect Dis. 2017 Jun 17;36(11):2101–7. doi: 10.1007/s10096-017-3032-6 (PMC5653722; doi:10.1007/s10096-017-3032-6)
Supplement: Supplementary file 1 — (DOCX 14 kb) [file 10096_2017_3032_MOESM1_ESM.docx]

**Supplement 1**

**Collection from the Institute of Tropical Medicine (ITM)**

The first collection (n=194) was obtained from symptomatic and asymptomatic travelers presenting for post-travel consultation at the ITM outpatient department between July 2007 and November 2013. Patients presenting at the HIV/STI policlinic were excluded, unless they had a recent travel history (n=1). Fresh stool samples were transported to the laboratory in sealed containers and processed within 24 hours after collection. The samples were diluted to a 1:5 g/ml saline suspension, inoculated on Campylobacter agar Butzler (Becton Dickinson GmbH, Heidelberg, Germany) and incubated in microaerophilic conditions generated by Anaerocult C (Merck Millipore, Darmstadt, Germany) at 42 °C for 24-48 hours. Identification of isolates was done using standard methods including colony morphology, Gram stain, catalase and oxidase reactions. Differentiation between *C.* *coli* and *C.* *jejuni* was done by testing for hippurate hydrolysis [1]. Subcultures were stored on Microbank (Pro-Lab Diagnostics, Cheshire, United Kingdom) at -80°C.

**Collection from the “Laboratoire de la Porte de Hal" (now : Laboratoire Hospitalier Universitaire de Bruxelles (LHUB-ULB), Site Porte de Hal.**

The second collection (n=84) was obtained from the Department of Microbiology of the “Laboratoire de la Porte de Hal” serving the Jules Bordet Institute (a hospital dedicated to cancer) and Saint-Pierre University Hospital in Brussels. At these hospitals, stool samples from outpatients suspected of gastroenteritis obtained from January 2008 to December 2013 were cultured for presence of *Campylobacter* spp. Only patients with a history of travel (≤2 weeks if symptomatic and ≤8 weeks if asymptomatic) prior to consultation were included in this study and only the first isolate per patient was considered. Stool samples were transported to the laboratory in clean and sealed containers or triple faeces test containers and usually processed within 4 hours, with a possible delay during weekends. A fecal suspension of approximately 1 g/ml saline was inoculated onto Butzler medium (Thermo Fisher Scientific, Erembodegem, Belgium) and incubated for 48 hours at 42°C in a microaerophilic atmosphere (CO_2_ 10%, O_2_ 5%, H_2_ 0%) with H2 optimization in case of suspected non jejuni or coli *Campylobacter* and examined daily for bacterial growth. In parallel, stool samples were diluted 1:5 in Brucella broth. Cellulose acetate filters, (0.45 µm pore size) were placed on the surface of non-selective Mueller Hinton agar plates containing 5% sheep blood. Eight drops of the fecal suspension were placed on top of the membrane and allowed to filter passively for 30 minutes at 37°C in a microaerophilic atmosphere.[2]. The filters were then removed and the plates were incubated at 37°C in a microaerophilic atmosphere for up to 10 days. Isolates were confirmed as *Campylobacter* spp. using standard biochemical methods and MALDI-TOF Mass Spectrometry (Vitek-MS (bioMérieux Marcy l'Etoile, France)) and were stored at -80°C on peptone water. For the purpose of this study, subcultures of all isolates obtained from patients with a reported travel history were shipped to ITM and stored on Microbank at -80°C pending testing.

**Collection from the Belgian National Reference Centre (NRC) for *Campylobacter* (National Survey)**

The third collection (n=37) consisted of isolates obtained between July and September 2013 during a nationwide survey study in Belgium comprising 92 laboratories. In each participating laboratory, the first 10 isolates obtained from routinely submitted stool samples from symptomatic patients were considered. Eighty-two laboratories used selective media (Campylosel selective agar (56.5% of laboratories), Butzler medium (21.7%) and Karmali medium (10.9%)). The isolation method was unknown in 10 cases (10,9%). Two laboratories reported the additional use of the filtration technique for the isolation of Campylobacter spp. (2). Approximately one third of the participating laboratories used MALDI-TOF Mass spectrometry for identification (either the Microflex LT (Bruker, Bremen, Germany) or Vitek-MS (bioMérieux)); the other laboratories used standard manual biochemical identification. Isolates were shipped to the NRC using FecalSwabs, then grown onto a Columbia agar containing 5% sheep blood and stored at -80°C on peptone water pending further analyses. For the current study only travel-related isolates were considered.

**References**

[1] Isenberg HD (2007) Aerobic bacteriology (section 3). In: Church DL (ed) Clinical microbiology procedures handbook

[2] Lopez L, Castillo F, Clavel A, Rubio M (1998) Use of a selective medium and a membrane filter method for isolation ofCampylobacter species from Spanish paediatric patients. European Journal of Clinical Microbiology and Infectious Diseases 17 (7):489-492
